# Complication Rates Following Septoplasty With Inferior Turbinate Reduction

**DOI:** 10.31486/toj.19.0002

**Published:** 2019

**Authors:** Rina R. Joshi, Charles A. Riley, Ashutosh Kacker

**Affiliations:** Department of Otolaryngology, Weill Cornell Medical College, New York, NY

**Keywords:** *Infection*, *nasal obstruction*, *nasal septum*, *perforations*, *septoplasty*, *turbinate reduction*

## Abstract

**Background:** Septoplasty with submucous resection of the inferior turbinate (SMRT) is a common correctional surgery performed in patients with deviated nasal septum resulting in nasal obstruction. Although complications are infrequent, studies examining long-term complications following septoplasty with SMRT are rare.

**Methods:** We conducted a retrospective review of patients electing to undergo septoplasty with SMRT at a tertiary rhinology clinic from January 2007 to December 2015. Demographic data, intraoperative findings, duration of follow-up, and short-term and long-term complications were collected. Exclusion criteria included patients who underwent either septoplasty or turbinate reduction or any other nasal surgery, patients lost to follow-up within 1 year, and patients with incomplete medical records.

**Results:** A total of 359 patients met inclusion criteria. The majority were males (66.6%), and the average age of the cohort was 36.8 ± 12.3 years. The mean follow-up time was 23.3 months. Short-term complications were postoperative infection (n=12, 3.3%) and epistaxis that required intervention (n=16, 4.5%). Long-term complications occurred in 10 patients (2.8%): revision septoplasty (n=9, 2.5%) and hyposmia (n=1, 0.3%). No instances of synechiae, septal perforation, or saddle nose deformity occurred.

**Conclusion:** Long-term complications following septoplasty with SMRT are infrequent. The most common long-term complication in this cohort was revision septoplasty.

## INTRODUCTION

Septoplasty with submucosal resection of the inferior turbinate (SMRT) is a common correctional surgery performed in patients with a deviated nasal septum and nasal obstruction. Approximately 20% of the general population has a deviated nasal septum, and 25% of these patients report difficulty breathing.^1^ Although the primary goal of septoplasty with SMRT is to decrease nasal obstruction and improve nasal airflow, the procedure may also be performed to alleviate symptoms such as epistaxis, headaches, and sinusitis.^2^ Overall, septoplasty with SMRT has been deemed a safe and effective procedure.^2^

Complications from septoplasty with SMRT are uncommon. Complications in the perioperative period include infection, nerve injury, and epistaxis. Previous studies have reported a postoperative infection rate of approximately 3% and a postoperative bleeding rate of nearly 6%.^3-5^ The majority of complications can be managed in the outpatient setting, although occasional admission and more aggressive treatment, such as intravenous antibiotics, may be necessary. Examples of complications that may require hospital admission are sepsis secondary to nasal packing with no antibiotics, profuse bleeding that persists despite pressure, and septal perforation.

Long-term complications, while known to occasionally occur, have not been rigorously studied. Such complications include failure to improve the nasal airway, resulting in revision surgery; saddle nose deformity; anosmia; cerebrospinal fluid (CSF) rhinorrhea; and blindness.^5^ CSF rhinorrhea is a rare but fatal complication of septoplasty that can occur as a result of fracturing the cribriform plate or tunneling into the ethmoid roof.^6^ This complication can be avoided by understanding anatomic variations in patients, especially in patients with a low-lying cribriform plate.^6^ In terms of other complications, Rao et al reported the case of a patient who experienced cranial nerve III palsy that resulted from retinal artery occlusion.^7^ The occlusion was presumed to be attributable to a retrograde flow of blood that was triggered via a combination of local anesthetic and adrenalin in the mucosa.^7^ In a case reported by Atighechi et al, the patient lost control of the medial rectus muscle (innervated by the third cranial nerve) as a result of radiofrequency ablation of the inferior turbinate.^8^

The goal of this study was to assess the prevalence of short-term and long-term complications after septoplasty with SMRT. A secondary aim of this study was to determine the rate of revision septoplasty.

## METHODS

### Patients

We conducted a retrospective review of patients aged 18 to 80 years who elected to undergo septoplasty with SMRT at a tertiary rhinology clinic from January 2007 to December 2015. All surgeries were performed under the care of the senior author (A.K.) at Weill Cornell Medical Center/New York Presbyterian Hospital. We reviewed patient medical records for date of surgery, sex, age, and complications. Short-term complications were defined as occurring within 2 weeks of surgery and included local infection or epistaxis and in rare cases nerve injury. Long-term complications were defined as occurring more than 2 weeks after surgery and included infection, septal perforation, epistaxis, and revision surgery.

Exclusion criteria included the performance of additional surgery at the time of septoplasty and turbinate reduction, if a patient was lost to follow-up, or if the initial septoplasty was performed by a surgeon other than the senior author. All patients had to have received inferior turbinate reduction in conjunction with septoplasty. This study was approved by the institutional review board of Weill Cornell Medical College.

### Procedure

The procedure was a standard septoplasty with cartilage replacement. Any perforations were noted via clinical symptoms or visualized on anterior rhinoscopy. All procedures were performed without endoscopic assistance. A modified Killian incision was used at the mucocutaneous junction. The cartilage was morselized and replaced. The nasal septal flaps were carefully elevated and preserved with careful reapproximation of the flaps with a coapting stitch. A 1 cm cartilaginous strut was preserved. The maxillary crest was removed using a V gouge. Mucosal sparing techniques were used to avoid perforation and saddle nose deformity. The incision sites were closed using chromic gut suture, and plain gut was used as a quilting suture. No packing was required or used. Splints were used if the patient had redundant mucosa that needed to be redraped. If splints were used, patients received 5 days of antibiotics. No clinically relevant septal hematomas were encountered.

## RESULTS

A total of 373 patients underwent septoplasty with inferior turbinate reduction. Eleven patients were lost to follow-up; 3 patients had undergone previous septoplasty prior to 2007 and were excluded. The cohort was comprised of 359 patients; demographic data are presented in Table 1. The standard postoperative follow-up schedule for routine patients undergoing septoplasty with SMRT is 7 days following surgery with a second follow-up visit 6 months later. The average follow-up time was 23.3 months with a range of 7 days to 7 years.

**Table 1.** Demographic Data of Patients Undergoing Septoplasty With Submucous Resection of the Inferior Turbinate (n=359)

Variable	Value
Males, n (%)	239 (66.6)
Females, n (%)	120 (33.4)
Mean age, years	36.8 (range, 18-65)
Length of follow-up,	23.3 months (range, 7 days-7 years)
months	

A total of 38 complications (10.6%) occurred, the majority of which were short term (28 of 359, 7.8%). Short-term and long-term complications are listed in Table 2. Short-term complications did not require a visit to the operating room (OR) and were handled in the outpatient clinic. One patient underwent 2 revision surgeries separated by 60 months, so each surgery was counted separately. No instances of septal perforation with clinical symptoms, saddle nose deformity, or synechiae were noted in this cohort.

**Table 2.** Complication Rates of Patients Undergoing Septoplasty With Submucous Resection of the Inferior Turbinate

Variable	Value
Total complications	38 (10.6)
Short-term complications	28 (7.8)
Infection	12 (3.3)
Epistaxis	16 (4.5)
Long-term complications	10 (2.8)
Hyposmia	1 (0.3)
Revision septoplasty	9 (2.5)

Note: Data are presented as n (%).

## DISCUSSION

Septoplasty with SMRT is a common surgical procedure performed to correct a deviated nasal septum. Approximately 260,000 cases are performed annually, making septoplasty with SMRT one of the most frequently performed surgeries by an otolaryngologist.^9^ Short-term complications from this surgery are well described and include infection, bleeding, and sensory impairment.^6^ However, long-term complications remain incompletely defined.

This retrospective case series demonstrated an overall complication rate following septoplasty with SMRT of 10.6%. Short-term complications occurred with greater frequency than long-term complications in our cohort, as nearly 75% of all complications were observed within the first 2 weeks after surgery. Although long-term complications occurred less frequently, they often required revision surgery.

Postoperative infection after septoplasty with SMRT occurs in a minority of patients. In a 1992 study by Yoder and Weimert, 5 of 1,050 patients (0.48%) developed an infection following septoplasty with no prior prophylactic antibiotics.^3^ In an earlier study (1980), the authors’ retrospective analysis of 210 patients showed that 5 patients (2.4%) developed an infection following septoplasty.^10^ Other researchers have demonstrated a higher rate of postoperative infections, up to 12% (12 of 100 patients) following septoplasty with SMRT.^11^ However, despite these infections in the immediate postoperative period, examination of the patients nearly 2 years after surgery did not demonstrate impairment in nasal air flow.^11^ Our study corroborates the published literature, as we noted a 3.3% rate of postoperative infections. The risk of infection is so low that researchers conclude that the use of preoperative antibiotics is unnecessary and ineffective in preventing postsurgical infections.^3,11^ Therefore, postoperative oral antibiotic treatment is sufficient for preventing or treating postsurgical infections.

Postoperative epistaxis following septoplasty with SMRT can be significant, requiring intervention in the clinic or OR and may be distressing to the patient, the patient's family, and the provider. The reported rate of postoperative epistaxis is approximately 6%.^5,12^ Bloom et al estimated that true hemorrhage rates range from 6% to 13.4% and sometimes require overnight observation.^6^ Our rate of epistaxis requiring intervention was 4.5%. Differences in hemorrhage rates are likely multifactorial and may be attributable to the procedure itself, septal incision, method of inferior turbinate reduction, surgeon-related differences, or the use/lack of use of nasal packing. In our cohort, nasal packing was not used in any of our patients at the completion of the procedure. However, Dubin and Pletcher found that packing vs nonpacking made no difference in bleeding rates.^13^ Reiter et al retrospectively studied 75 patients who underwent septorhinoplasty with a quilting suture and absence of nasal packing.^14^ Of the 75 patients, only 2 experienced postoperative bleeding.^14^ Bleeding rate differences can also result from poor injection technique of vital septal blood vessels and incidental mucosal trauma.^6^

Long-term complications were less commonly encountered in our cohort, comprising 26.3% (10/38) of all complications. Persistent nasal obstruction requiring revision was the most common long-term complication. The etiology of this finding is not straightforward, as nasal obstruction is a subjective sensation. Bohlin and Dahlqvist found that patients who needed a revision septoplasty were experiencing persistent obstruction.^15^ Dommerby et al found that 23 of 161 patients felt that insufficient septal surgery hindered their long-term ability for nasal relief.^16^ Jessen et al found an increase in the percentage of patients complaining of nasal obstruction as they progressed from a 9-month to 9-year follow-up period.^17^ The authors proposed that as time progresses, patients no longer experience a sensation of relief from nasal obstruction and therefore regress to feeling congested.^17^

Bohlin and Dahlqvist found that 6.3% of patients required revision surgery.^15^ In a study conducted by Becker et al, 70 of 547 patients underwent a revision septoplasty (12.8%).^18^ Dinis and Haider sent postsurgical surveys to 135 patients who underwent septoplasty. Among the 79 patients who responded, only 1 required revision septoplasty.^19^ Dissimilarity in revision rates between studies could be attributable to the fact that the surgeons who performed the operations in the Bohlin and Dahlqvist study were still training.^15^ The difference in revision rates between the Dinis and Haider study and our study could be attributable to the manner in which data were collected.^19^ In our study, the revision rate was 2.5%, which is lower than most of the previous studies, perhaps because of the senior attending who performed the surgery, but higher than the rate reported by Dinis and Haider, perhaps because we collected data from patients’ medical records rather than via postsurgical surveys.

Interestingly, no examples of septal perforation, saddle nose deformity, or synechiae were noted upon long-term clinical follow-up. This finding is likely a limitation of the retrospective nature of our study, as the septal perforation rate following septoplasty is approximately 0.9%.^20^ Still, this finding suggests that meticulous elevation and preservation of the nasal septal flaps, careful reapproximation of the septal flaps with a coapting stitch, preservation of a 1 cm cartilaginous strut, and mucosal-sparing techniques are critical to avoid perforation and saddle nose deformity while performing septoplasty with SMRT.

Limitations of this study include a large stratification of the age group. Future studies should examine differences between specific age groups. Additional limitations included lack of patient input in regard to the effectiveness of the procedure. Because of the concurrent nature of the procedures in all patients included in this study, we could not determine which complications were the result of SMRT and which were attributable to septoplasty. Furthermore, the effects of comorbidities on complication rates and factors that increase postoperative bleeding, such as medication use, require investigation. Future studies might consider performing nasal endoscopy during long-term follow-up examinations to assess for septal perforations with clinical symptoms or other intranasal pathology not appreciated on anterior rhinoscopy. Additionally, utilization of the Nasal Obstruction Symptom Evaluation quality of life questionnaire is warranted to assess the long-term patient-reported outcomes and to examine how these symptoms evolve over time.^21^

## CONCLUSION

Septoplasty with SMRT is a common surgical procedure with several possible complications. Long-term complications occurring more than 2 weeks after surgery were infrequently encountered in this case series. The most common long-term complication was revision septoplasty. Therefore, we conclude that septoplasty is a relatively safe procedure that should be recommended for patients with a deviated nasal septum or other sinus issues. The minimal long-term complications, including revision septoplasty, demonstrate that most initial septoplasties are successful.

## Notes

This article meets the Accreditation Council for Graduate Medical Education and the American Board of Medical Specialties Maintenance of Certification competencies for Patient Care and Medical Knowledge.
